# Synthetic surfactant- and cross-linker-free preparation of highly stable lipid-polymer hybrid nanoparticles as potential oral delivery vehicles

**DOI:** 10.1038/s41598-017-02867-x

**Published:** 2017-06-05

**Authors:** Taoran Wang, Jingyi Xue, Qiaobin Hu, Mingyong Zhou, Chao Chang, Yangchao Luo

**Affiliations:** 1 Department of Nutritional Sciences, University of Connecticut, Storrs, CT 06269 USA; 2 School of Food Science and Engineering, Wuhan Polytechnic University, Wuhan, Hubei 430023 China

## Abstract

The toxicity associated with concentrated synthetic surfactants and the poor stability at gastrointestinal condition are two major constraints for practical applications of solid lipid nanoparticles (SLN) as oral delivery vehicles. In this study, a synthetic surfactant-free and cross-linker-free method was developed to fabricate effective, safe, and ultra-stable lipid-polymer hybrid nanoparticles (LPN). Bovine serum albumin (BSA) and dextran varying in molecular weights were first conjugated through Maillard reaction and the conjugates were exploited to emulsify solid lipid by a solvent diffusion and sonication method. The multilayer structure was formed by self-assembly of BSA-dextran micelles to envelope solid lipid via a pH- and heating-induced facile process with simultaneous surface deposition of pectin. The efficiency of different BSA-dextran conjugates was systematically studied to prepare LPN with the smallest size, the most homogeneous distribution and the greatest stability. The molecular interactions were characterized by Fourier transform infrared and fluorescence spectroscopies. Both nano spray drying and freeze-drying methods were tested to produce spherical and uniform pectin-coated LPN powders that were able to re-assemble nanoscale structure when redispersed in water. The results demonstrated the promise of a synthetic surfactant- and cross-linker-free technique to prepare highly stable pectin-coated LPN from all natural biomaterials as potential oral delivery vehicles.

## Introduction

Since introduced in 1991, solid lipid nanoparticles (SLN) have been one of most popular nanoscale delivery vehicles among many colloidal systems, such as protein- and polysaccharide-based nanoparticles, nanoemulsions, liposomes, and polymeric micelles^1^. SLN are usually prepared with saturated fatty acids (e.g. stearic acid) or physiological lipids (e.g. triglycerides), by solvent diffusion, microemulsion, hot/cold homogenization, and high pressure homogenization techniques^2^. Due to the hydrophobic core, lipophilic compounds can be easily loaded into SLN by dissolving in the organic phase with lipids during fabrication process, and their release mechanisms have been extensively studied for decades. Most of these applications are generally limited to parenteral administration, where the drug-loaded SLN are either injected intramuscularly, intravenously or subcutaneously^3^. Through all of these efforts, SLN have been demonstrated to be biocompatible and biodegradable with excellent tolerability and minimal toxicity. Thus, during the last decade, several studies began to explore the oral delivery potentials of SLN as bioavailability enhancer for drugs^4^ and particularly food bioactive components^5^.

Nevertheless, many studies neglected an important factor, that is, the physical stability of solid lipid matrix in the gastrointestinal (GI) tract. The nanostructure of solid lipid is extremely sensitive to acidic condition due to the severe agglomeration, resulting from the protonation of carboxyl groups of lipid molecules. Hence, SLN form large aggregates once entering the stomach through oral route, dramatically compromising their delivery efficacy in the GI tract. Thus far, several techniques have been reported to effectively prevent their agglomeration in stomach, such as PEGylation^6, 7^ and coating with high concentration of synthetic surfactants^8, 9^. These strategies, however, are associated with potential toxicity and production of cytokines^10^, and therefore drastically limit their clinical applications. Another major challenge of applying SLN clinically is to obtain redispersible dry powders without altering their nanostructure, so as to maximally extend their shelf life with low cost. Both freeze- and spray-drying methods have been attempted to obtain SLN dry powder^2^. Unfortunately, the slow rate of water removal during freeze drying and high temperature involved in spray drying are known to induce severe aggregation of SLN and powders with bulky structure (>10 µm) are usually obtained, unless a very high concentration of spacing agent, such as trehalose up to 30%^11^, is pre-added into the formulation. The inclusion of such a high concentration of sugar will undoubtedly jeopardize their practical applications.

Therefore, scientists have been exhaustively seeking for natural biomaterials to prepare and stabilize SLN without use of synthetic surfactants, and meanwhile testing novel drying technologies to obtain ultrafine redispersible SLN without addition of spacing agents. Recently, our laboratory has developed a novel strategy to simultaneously address the above two concerns. First, we demonstrated the feasibility to use natural biomacromolecules, including proteins and polysaccharides, to emulsify and prepare SLN without the need of synthetic surfactants^12, 13^. The proteins and polysaccharides serve as multilayer coatings to stabilize SLN from aggregation in GI conditions. We also reported that uniform, ultrafine and spherical lipid-based powder particles can be produced by the innovative Nano Spray Drying technology, when appropriate polymeric coatings were applied^12, 14^.

**Figure 1:**
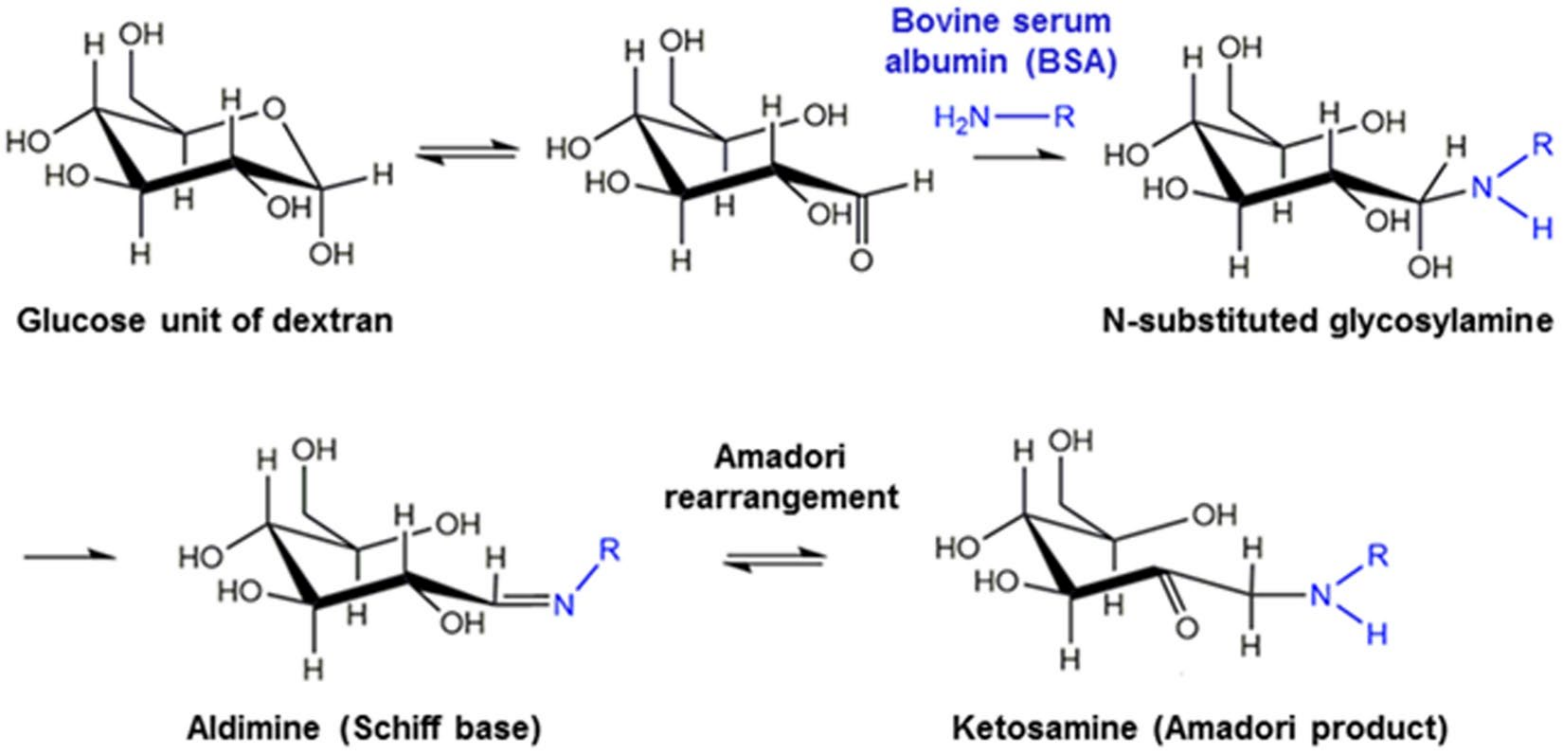
Schematic representation of Maillard conjugation reaction between glucose unit in dextran and bovine serum albumin (BSA).

In our recent work, we have discovered that chemical cross-linking of polymeric multilayer coatings, by creating either imine or amide bonds, is necessary to achieve acceptable stability under GI conditions^13^. Given the fact that chemical cross-linkers are usually associated with potential toxicity, it would be more desirable and practical for clinical applications if a cross-linker-free process can be developed to prepare stable SLN. Therefore, our present work investigated the feasibility of using Maillard reaction, as a green chemical reaction, to create amide bonds between the multilayer coatings formed by bovine serum albumin (BSA) and dextran to stabilize SLN. In principle, amino group of a protein reacts with carbonyl group of a reducing carbohydrate, i.e. BSA and dextran in the present study, producing N-substituted glycosylamine and then becomes Schiff base. The obtained unstable product undergoes an Amadori rearrangement, leading to a formation of ketosamine (1-amino-1-deoxy-ketose)^15–17^, which is also known as the Amadori product (Fig. 1). Although Maillard reaction conjugates have been widely reported to improve the physical stability of conventional emulsions^18–20^ and protein nanoparticles^15, 21^, their emulsification and stabilization effects on solid lipid nanoparticles have not been explored yet. In particular, we explored the potential of Maillard reaction products between dextran and BSA to emulsify solid lipid in this study. The obtained biopolymer-coated SLN were hereafter denoted as lipid-polymer hybrid nanoparticles (LPN). The potential of Maillard reaction products together with pectin as multilayer coatings to stabilize LPN under gastrointestinal conditions was investigated. The physicochemical properties of as-prepared LPN were comprehensively characterized and different drying technologies were also tested to produce spherical and redispersible powder for future applications as oral delivery vehicles.

## Experimental Details

### Materials

Compritol® 888 ATO was a gift from Gattefossé. BSA, dextran (molecular weight 20, 40, 75, 150, and 500 kDa) and pectin (galacturonic acid content ≥ 74%) were supplied by Sigma-Aldrich (St. Louis, MO, USA). Hydrochloric acid, sodium hydroxide, acetone, ethanol, and potassium bromide were obtained from Fisher Scientific Co. (Norcross, GA, USA). Unless noted otherwise, all chemicals were of analytical grade and used without further purification.

### Preparation of BSA-dextran Maillard conjugates

Maillard reaction was conducted according to previously reported protocols^22, 23^ with slight modifications. Briefly, BSA and dextran (with different molecular weight) stock solutions were respectively prepared by dissolving in ultrapure water at 40 mg/mL and 100 mg/mL. A dextran stock solution was mixed with 1.25 mL of 40 mg/mL BSA solution dropwise under gentle stirring to reach the final 2.5 mg/mL concentration of BSA and total volume of 20 mL in each group. The molar ratio of protein to dextran in the mixture was in the range of 1:4 to 4:1. The pH of the mixture was adjusted to 8.0 using 0.1 mol/L NaOH, followed by the lyophilization for 48 h in a Labconco FreeZone 6 Freeze Dry System (Kansas City, MO, USA). The freeze-dried powder was incubated under 79% relative humidity in a desiccator containing saturated potassium bromide solution for 24 h at 60 °C. The reaction products were denoted as BSA-dextran conjugates and were used to prepare LPN. The detailed sample information in different groups is tabulated in Table 1.

**Table 1:** Formulations for preparation of Maillard conjugates (^a^ MR: Molar ratio).

Group	Dex20	Dex40	Dex75	Dex150	Dex500	MR^a^ of BSA to dextran
M.W. of dextran (kDa)	20	40	75	150	500	
Subgroup	Dex20-1	Dex40-1	Dex75-1	Dex150-1	Dex500-1	1:4
	Dex20-2	Dex40-2	Dex75-2	Dex150-2	Dex500-2	1:2
	Dex20-3	Dex40-3	Dex75-3	Dex150-3	Dex500-3	1:1
	Dex20-4	Dex40-4	Dex75-4	Dex150-4	Dex500-4	2:1
	Dex20-5	Dex40-5	Dex75-5	Dex150-5	Dex500-5	4:1

### Sodium dodecyl sulfate-polyacrylamide gel electrophoresis (SDS-PAGE)

The reducing SDS–PAGE was carried out to confirm the formation of conjugates between BSA and dextran. A 5 µL BSA-dextran conjugate (containing 2.5 µg of BSA before Maillard reaction in each group) or 0.5 mg/mL BSA solution dissolved in a 0.01 M phosphate buffer (pH 7.4) was mixed with 4.75 µL Laemmli sample buffer and 0.25 µL β-mercaptoethanol. The above mixture was then heated at 100 °C for 5 min and loaded onto the gel. Electrophoresis was performed at 200 V for about 1 hr with a 0.1% Tris–glycine electrophoresis buffer. The gel was stained by Coomassie Brilliant Blue solution for 1 h and rinsed in 200 mL ultrapure water for about 30 min. The gels were then scanned and pictured using a scanner.

### TNBS assay

2,4,6-Trinitrobenzene Sulfonic Acid (TNBS) spectrophotometric assay is a fast, sensitive, and convenient assay to determine grafting degree of BSA by detecting the free amino groups in the Maillard conjugates. The TNBS assay reagent was prepared according to Hermanson *et al*.^24^. First, the conjugates were dissolved at a concentration of 200 µg/mL with reaction buffer (0.1 M sodium bicarbonate, pH 8.5). BSA solutions with a series of dilutions ranging from 0 to 200 µg/mL in reaction buffer were prepared for standard calibration curve. Then, 0.25 mL of 0.01% (w/v) TNBS solution was added and mixed with 0.5 mL of each sample and standard solution, followed by 2 h incubation at 37 °C. The TNBS solution was prepared and diluted freshly every time prior to use. After incubation, 0.25 mL 10% (w/v) SDS solution (diluted with distilled water) and 0.125 mL of 1 M HCl solution were immediately added into each sample, and then the absorbance at 335 nm was measured. The grafting degree of BSA was calculated using following equation:

$${\bf{Grafting}}\,{\bf{degree}}\,\,({\bf{GD}})=\frac{{\bf{Concentration}}\,{\bf{of}}\,{\bf{free}}\,{\bf{amino}}\,{\bf{groups}}\,{\bf{in}}\,{\bf{BSA}}\,{\bf{detected}}\,{\bf{in}}\,{\bf{sample}}}{{\bf{Concetration}}\,{\bf{of}}\,{\bf{total}}\,{\bf{amino}}\,{\bf{groups}}\,{\bf{in}}\,{\bf{BSA}}\,{\bf{added}}\,{\bf{in}}\,{\bf{sample}}}\ast \mathrm{100}{\boldsymbol{ \% }}$$

### Preparation of LPN using BSA-dextran conjugates and pectin coating

**Figure 2:**
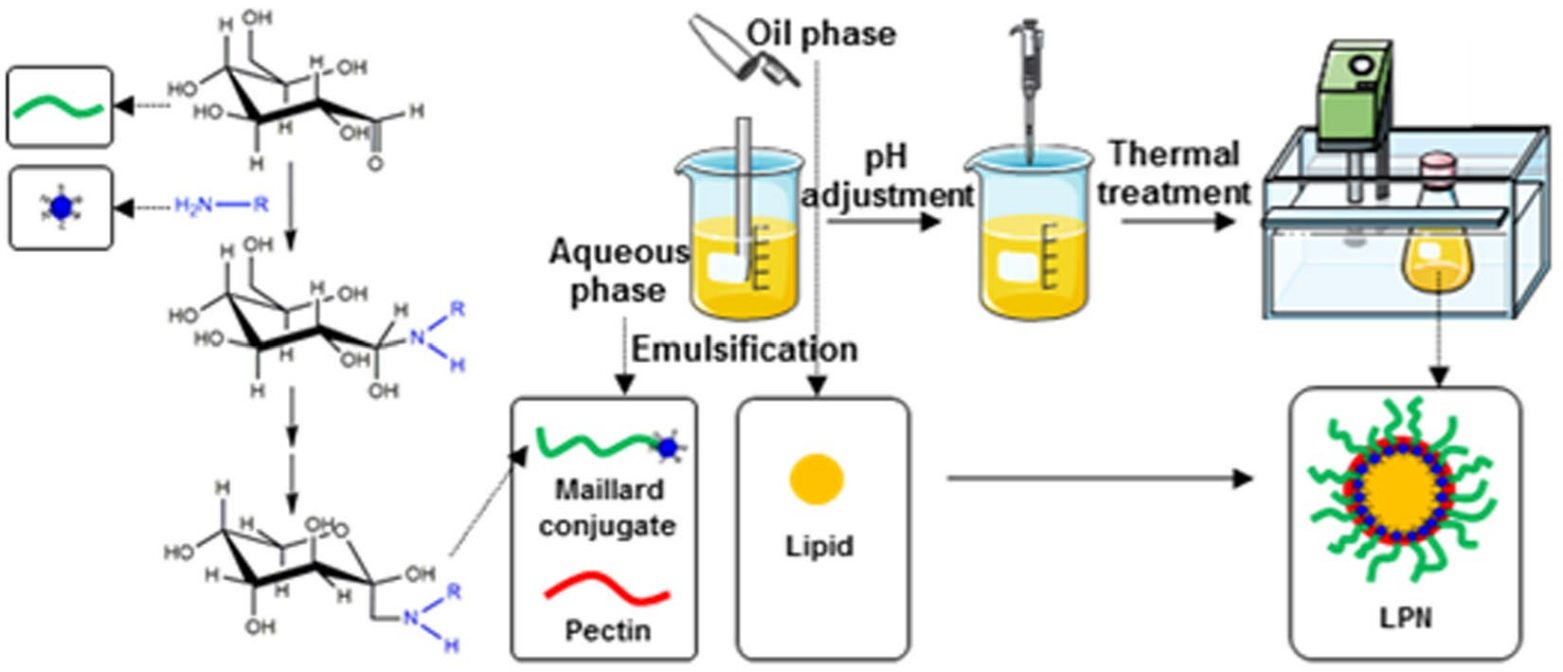
Schematic diagram of fabrication of pectin-coated LPN.

LPN was prepared by emulsifying solid lipid with various BSA-dextran conjugates via solvent-diffusion and sonication combined technique, as modified from our previous study^25^. Briefly, 10 mg of Compritol® 888 ATO was completely dissolved in the organic phase (0.5 mL acetone and 0.5 mL ethanol), which was preheated to 80 °C in a water bath. The obtained organic phase was added into 10 mL of aqueous phase containing BSA-dextran conjugates at 1 mg/mL, followed by 3 min sonication by a probe-type sonicator (Misonix Sonicator^®^ 3000, USA). Then, the samples were rapidly cooled down in an ice bath to solidify the lipid nano-droplets. To prepare pectin-coated LPN, a pH- and heat-induced deposition process was adopted, as previously described in our recent work^12^. Briefly, pectin was dissolved in ultrapure water at 2 mg/mL and hydrated overnight, followed by pH adjustment to 6.8. The above pectin solution was then added into the aqueous phase (BSA-dextran conjugates solution), followed by 3 min sonication. The pH of the mixture was adjusted to pH 4.7 and then heated at 80 °C for 30 min to reinforce the polymeric coating network. After that, samples were rapidly cooled down in ice bath to solidify lipid core and so the LPN was obtained. The coating procedures are illustrated in Fig. 2. Nanoparticle controls were prepared similarly but using BSA and/or dextran instead of BSA-dextran conjugates. The detailed information of control groups was summarized in Table 2.

**Table 2:** Formulations for control groups.

	Control group	Lipid (mg)	BSA (mg)	Dextran (mg)	Pectin (mg)	V (mL)
C1	BSA	10	10	—	—	10
C2	BSA w/pectin	10	10	—	5	10
C3	Dextran, 75k	10	—	10	—	10
C4	Dextran, 75k w/pectin	10	—	10	5	10
C5	BSA, dextran mixture	10	4.7	5.3	—	10
C6	BSA, dextran mixture w/pectin	10	4.7	5.3	5	10

### Characterization of LPN

The particle size, polydispersity index (PDI), and zeta potential of LPN were measured by a Zetasizer Nano ZS at 25 °C (Malvern Instruments Ltd, Worcestershire, UK). The hydrodynamic dimeter (particle size) and PDI were determined by Dynamic Light Scattering (DLS) at a scattering angel of 173°. All samples were diluted 10 times with ultrapure water to fit the optimal instrument sensitivity and avoid multiple scattering. To record the comparable count rate data, all samples were measured with a fixed attenuator. Zeta potential was calculated from electrophoretic mobility measured by a laser Doppler velocimeter using the same instrument.

Colloidal LPN samples were cast-dried on an aluminum pan and stored in a vacuum desiccator prior to Fourier transform infrared (FT-IR) spectrum analysis by a Nicolet iS5 FT-IR spectrometer (Thermo Scientific, Waltham, MA, USA). The infrared spectra were collected from the wavenumber of 500–4000 cm^−1^ at a resolution of 4 cm^−1^ and analyzed using OMNIC software version 8.0.

To determine the gastrointestinal stability of as-prepared LPN, 1 mL of sample was added to 9 mL of simulated gastric fluid (pH 2 or 4, with 1 mg/mL pepsin) and incubated at 37 °C for 2 h. After that, 1 mL of the above mixture was mixed with 9 mL of simulated intestinal fluid (pH 7 with 10 mg/mL pancreatin) and incubated at 37 °C for 4 h. To minimize the scattering effect of undissolved prancreatin, it was hydrated overnight, centrifuged, and filtered through 0.45 µm membrane to remove any insoluble impurities prior to use. After each incubation, the particulate characteristics, including particle size, PDI and zeta potential, were determined as described above.

### Fluorescence spectroscopy

The fluorescence spectra of native BSA, BSA-dextran conjugates (Dex20–3, Dex40-3, Dex75-3, Dex150-3, and Dex500-3), LPN (prepared with conjugate Dex75-3), and pectin-coated LPN (prepared with conjugate Dex75-3) were recorded using a LS55 Fluorescence Spectrofluorometer (PerkinElmer, Waltham, MA, USA). The excitation wavelength was 280 nm, and the emission spectra were collected by scanning from 300 to 500 nm. The step width was set at 0.5 nm for both excitation and emission. All samples were diluted with ultrapure water in order to meet the instrument sensitivity range. For native BSA and BSA-dextran conjugates, they were dissolved at 1 mg/mL in ultrapure water as the stock solution and then diluted to the same final concentration as in the LPN samples.

### Drying and redispersion

Selected pectin-coated LPN samples were dried by either a Nano Spray Dryer B-90 (Büchi Labortechnik AG, Flawil, Switzerland) or a Labconco FreeZone 6 Freeze Dry System (Kansas City, MO, USA). For nano spray drying, the following operating conditions were performed: inlet temperature at 100 °C, flow rate at 120 L/min, and mesh size of 5.5 µm. For freeze-drying, samples were first frozen at −80 °C overnight and then freeze-dried at −80 °C/0.014 mBar for 24 h. The obtained sample powders were checked for their redispersibility by dissolving powders in water at a concentration of 1 mg/mL and heating at 80 °C for 5 min was applied to achieve better redispersibility. The particle size, PDI, and zeta potential were determined by DLS technique and the morphology of powders as well as the redispersed LPN samples were observed under SEM as described in the following section.

### Morphological observation

Transmission electron microscopy (TEM) was used to observe the morphology of freshly prepared pectin-coated LPN (Dex75-3). Samples were stained by mixing with equal volume of 2% phosphotungstate (pH adjusted to 7), and then one drop of stained sample was deposited on a 400-mesh copper grid for 10 min, prior to morphological observation using a TEM (Tecnai T12, FEI, Hillsboro, Oregon, USA) at 80 kV. Scanning electron microscopy (SEM) was conducted to visualize the morphology and shape of spray-dried, freeze-dried LPN powders and redispersed particles. For powders, sample was directly placed on double-sided carbon tape pre-affixed on a specimen stub, and for redispersed samples, a few drops of sample were cast-dried on an aluminum pan overnight and then placed on the double-sided carbon tape pre-affixed on a specimen stub. Both powder and redispersed samples were sputter-coated with a thin layer of gold before observed under SEM (JSM-6335F, JEOL Ltd., Tokyo, Japan).

### Statistical analysis

All experiments were conducted in triplicate at least. The results were expressed as the mean ± standard deviation. One-way ANOVA with Tukey’s multiple-comparison test was used to compare the significance among samples. P-value less than 0.05 was considered to be statistically significant.

## Results and Discussion

### Formation of BSA-dextran conjugates by Maillard reaction

**Figure 3:**
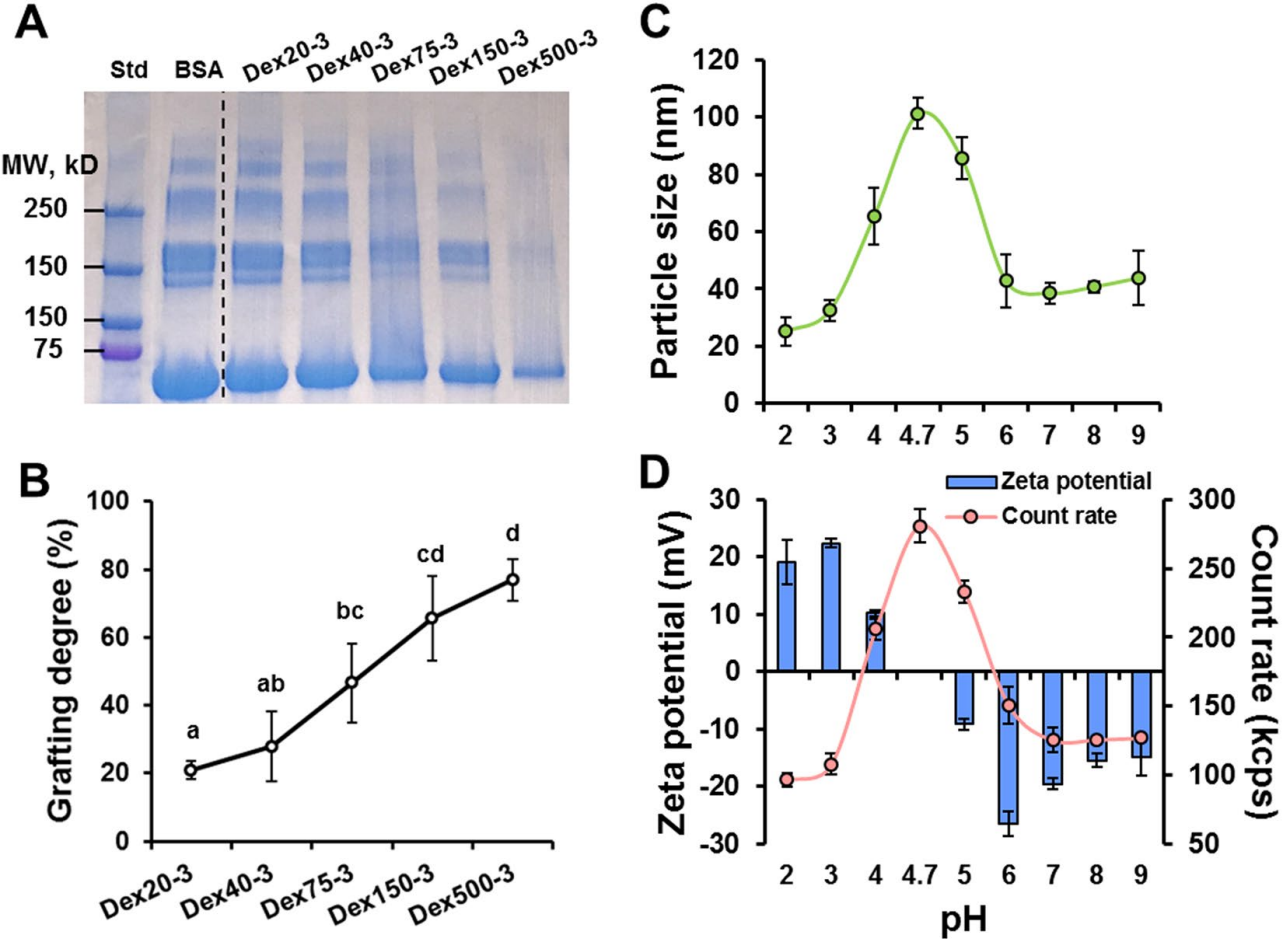
(**A**) SDS–PAGE analysis of BSA-Dextran conjugates produced by Maillard reaction with different experimental groups; (**B**) Grafting degree of Maillard conjugates; the pH dependence of particle size (**C**), and count rate and zeta potential (**D**) of Maillard conjugate (Dex75-3). In Fig. 3B, data not sharing the same letter were significantly different (p < 0.05).

The molecular weight profile of native BSA and five conjugates (Dex20-3, Dex40-3, Dex75-3, Dex150-3, and Dex500-3) are analyzed by SDS-PAGE and shown in Fig. 3A. The native BSA molecule showed a strong and clear band at 66 kDa, corresponding to the molecular weight of native BSA. In addition, the native BSA also exhibited several smear bands at higher molecular weights ranging from 150 to 300 kDa, which could be due to the impurities or aggregates of BSA monomers during heating process of sample preparation for SDA-PAGE^26–28^. Maillard reaction between BSA and dextran had a prominent effect on the molecular weight profile. Gradual and significant reduction in the intensity of native BSA characteristic bands was observed with the increase of dextran molecular weight (Fig. 3A). Notably, there was only one visible band at 66 kDa in the sample Dex500-3, and a significant amount of sample was observed on the top of the separating gel, suggesting the formation of high molecular weight BSA-dextran conjugate. Figure 3B showed the grafting degree of five Maillard conjugates measured by TNBS assay. In TNBS assay, free primary amines on BSA reacted with TNBS forming a highly chromogenic derivative. As the increase of dextran molecular weight, the grafting degree of conjugates increased from about 30% to 77%, confirming that the higher molecular weight dextran, which has longer molecular chain containing more carbonyl groups, was more effective to consume amino groups on BSA. This result well agreed with SDS-PAGE data in Fig. 3A. The capability of BSA-dextran Maillard conjugates to form micelle structure was studied as a function of pH values (Fig. 3C and D). The particle size, count rate, and zeta potential were measured by DLS to monitor the formation of micelles. At acidic (pH 2–3) or basic (pH 7–9) conditions that are away from BSA’s isoelectric point (pI, pH 4.7)^29^, the BSA moiety carries strong positive or negative charges, respectively, and so the conjugate exists as monomeric protein molecules with strong repulsive forces among monomers. Concomitantly, the count rate at such conditions was very small, representing the concentration of colloidal particles was low. In contrast, at range of pH 4–5, which is close to the pI of BSA, there was a significant raise in the count rate and particle size reached 100 nm at pH 4.7 with the highest count rate and zero zeta potential. This was indicative of that BSA moieties in the conjugates aggregated together to form micelles while the attached dextran tail stabilized the micelles from precipitation. Our observation on the formation of BSA-dextran micelles at acidic condition was well corroborated by other literature reports^30, 31^.

### Characterization of LPN prepared with different BSA-dextran conjugates

**Figure 4:**
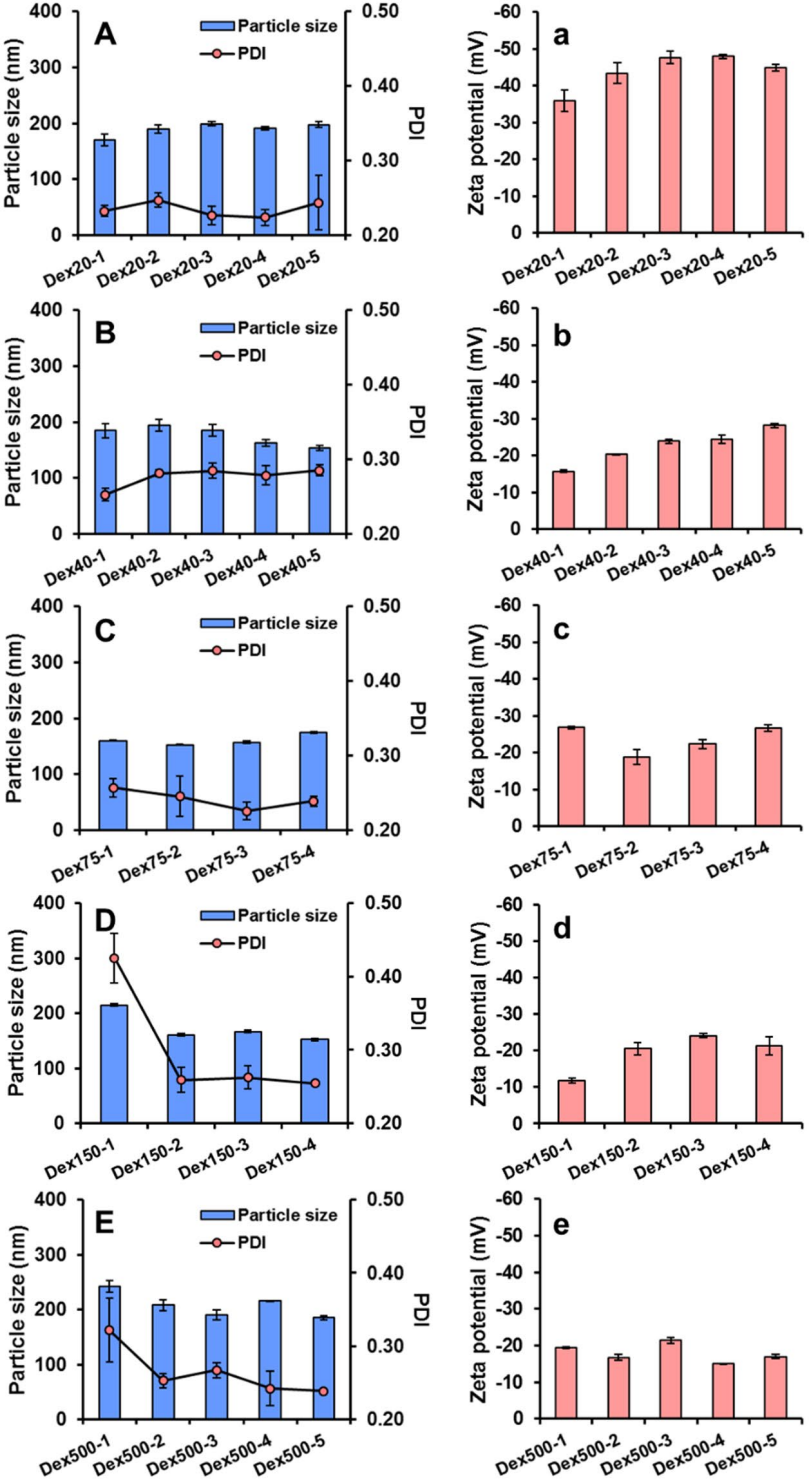
Particle size, PDI (**A–E**), and zeta potential (a–e) of LPN (prior to pectin coating) prepared using Maillard conjugates with different molecular weight dextran. The groups Dex75-5 and Dex150-5 formed gels and were not completely solubilized in water, and thus their results were excluded.

The particulate characteristics, including particle size, PDI and zeta potential of LPN prepared with different BSA-dextran conjugates are depicted in Fig. 4. It was noticed that the mass ratio between BSA and dextran in the conjugates did not show significant effects on the particle size and PDI. Generally, the particle size and PDI of LPN were in the range of 150–200 nm and 0.2–0.25, respectively, except samples Dex150-1 (Fig. 4D) and Dex500-1 (Fig. 4E), which exhibited greater particle size (>200 nm) with larger PDI values (>0.3). This may be partly due to the larger molecular weight and greater molar ratio of dextran in the conjugates, resulting in the very thick and heterogeneous polymeric coating and polydispersed particles. In contrast, the zeta potential of LPN, directly related to the net charges on the surface of nanoparticles, was affected by the types of conjugates to a greater extent. When the smallest molecular weight of dextran was used to form the conjugates, the prepared LPN (Fig. 4a) carried stronger negative surface charge, explaining the substantial exposure of carboxyl groups of BSA on the nanoparticles surface due to the limited shielding effect from conjugated dextran. As the increase of dextran molar ratio in the conjugates, the shielding effect from dextran became more prominent and the zeta potential of LPN gradually reduced (Fig. 4a–e). Concomitantly, among the LPN prepared by the conjugates with the same molecular weight dextran, the magnitude of zeta potential was generally increased with the decrease of dextran molar ratio. It is worth noting that the particle size and PDI of LPN prepared by this synthetic surfactant-free method were similar to or even smaller than many reported SLN systems prepared with concentrated synthetic emulsifiers/surfactants^32–34^.

### Adsorption of pectin onto LPN by electrostatic deposition

**Figure 5:**
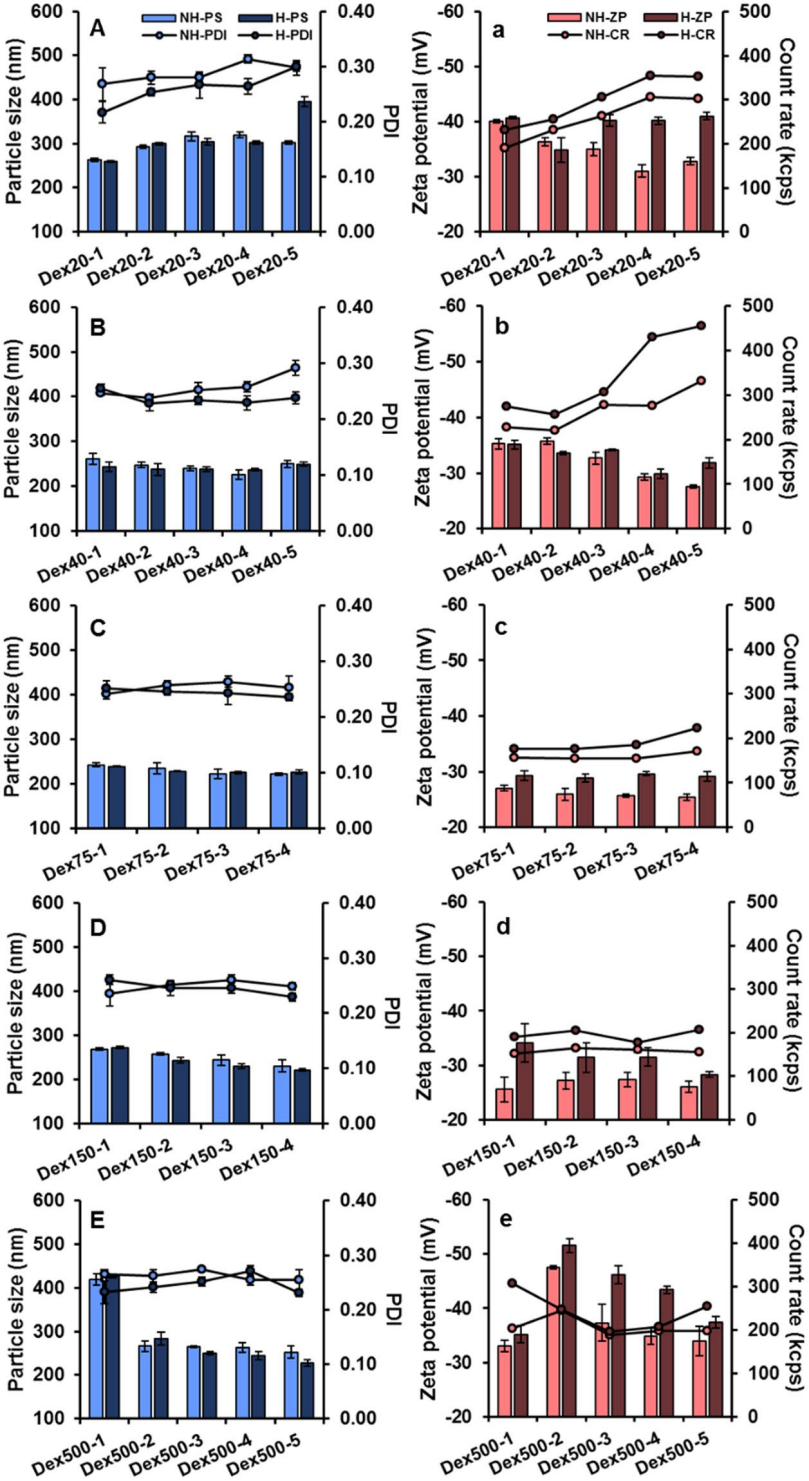
Particle size, PDI (**A–E**), zeta potential, and count rate (a–e) of pectin-coated LPN (before and after thermal treatment) prepared using Maillard conjugates with different molecular weight dextran. The groups Dex75-5 and Dex150-5 formed gels and were not completely solubilized in water, and thus their results were excluded. NH: non-heat, H: heat, PS: particle size, ZP: zeta potential, CR: count rate.

Although the BSA-dextran conjugates were stable at a wide range of pH and formed micelles upon heating at pH 4.7 (Fig. 3), our preliminary data showed that the prepared LPN severely aggregated and precipitated at gastric condition (pH 2–4). This revealed that conjugates alone could not protect carboxyl groups of lipid molecules from protonation at acidic pHs. Therefore, to improve their physical stability at acidic pHs, we introduced pectin as the third polymeric coating layer on the LPN and tested whether heating the pectin-coated LPN at pH 4.7 would reinforce the coating structure. It was apparent that the particle size of LPN increased by 50–100 nm after pectin coating, while heating process did not have a significant impact even though a slight reduction on particle size was noticed (Fig. 5). Similar trends were observed for PDI that the heated LPN had a smaller PDI, indicating the formation of a more compact and homogeneous network upon heating. The magnitude of zeta potential of LPN after pectin coating generally increased by 15–20 mV, except the samples in group Dex20 (Fig. 5a) where a slight reduction was observed. Moreover, heating process further increased the magnitude of zeta potential by approximately 5–10 mV. Count rate, a reflection of particle density in a colloidal system, increased significantly by the heating process (Fig. 5a–e), revealing that pectin adsorption had occurred and the strong interaction between pectin and BSA-dextran layers resulted in the formation of more compact LPN.

Heating protein/polysaccharide mixture at a pH close to the protein’s pI is a well-known process to induce partial denaturation and self-aggregation of protein molecules and simultaneous deposition of polysaccharide molecules, and thus the protein/polysaccharide complex nanoparticles were formed^35, 36^. Our previous study showed that casein/pectin nanocomplex particles prepared by this process can be not only applied as an oral delivery vehicle^37^, but also holds great potential to coat LPN^12^. The LPN in our present study exhibited much smaller particle size than casein/pectin-coated SLN (>350 nm) in our previous study^12^, which may be explained by the following two major reasons. First, the protein/polysaccharide Maillard products often possess significantly improved emulsifying properties than their physical mixture^16^, and so the solid lipids are better emulsified during preparation. Second, the high-energy sonication is used in the present study to reduce the particle size, compared to the high-speed homogenization technique in our previous work. In addition, the increased magnitude of zeta potential and count rate with the same particle size after heating is indicative of the internal structural change and the re-arrangement of pectin network on the outmost surface of LPN, confirming the formation of denser and more compact polymeric coating^37, 38^. In general, BSA-dextran conjugates with medium molecular weight (75 and 150 kDa) exhibited smaller particle size and PDI, as well as stronger interactions with pectin as indicated by the greater extent of increase in zeta potential upon heating. Thus, they are considered as the optimal formulations for production of LPN. Additionally, in order to justify the coating effect of pectin and BSA/dextran conjugate, various control groups (Table 2) were conducted and the results were tabulated in Table 3. Although all control groups showed a relative small particle size and PDI at pH 7, they all precipitated after pH was adjusted to 4.7 and thermal treatment. These results indicated that BSA or dextran alone, or their physical mixture, could not protect lipid nanoparticles under acidic environment.

**Table 3:** Particle size, PDI, and zeta potential of control groups under different pH and condition.

Group	pH 7			pH 4.7	pH 4.7 w/thermal treatment
	Particle size (nm)	PDI	Zeta potential (mV)		
C1	145.1 ± 0.3	0.284 ± 0.018	−18.6 ± 0.7	Precipitates	Precipitates
C2	157.4 ± 1.5	0.292 ± 0.018	−35.9 ± 1.5	Precipitates	Precipitates
C3	140.5 ± 2.2	0.272 ± 0.014	−28.6 ± 0.5	Precipitates	Precipitates
C4	174.5 ± 1.2	0.270 ± 0.006	−34.6 ± 1.1	Precipitates	Precipitates
C5	169.2 ± 1.5	0.286 ± 0.010	−25.1 ± 0.8	Precipitates	Precipitates
C6	217.1 ± 1.6	0.259 ± 0.015	−36.0 ± 0.3	Precipitates	Precipitates

### Stability of LPN in simulated gastrointestinal conditions

**Figure 6:**
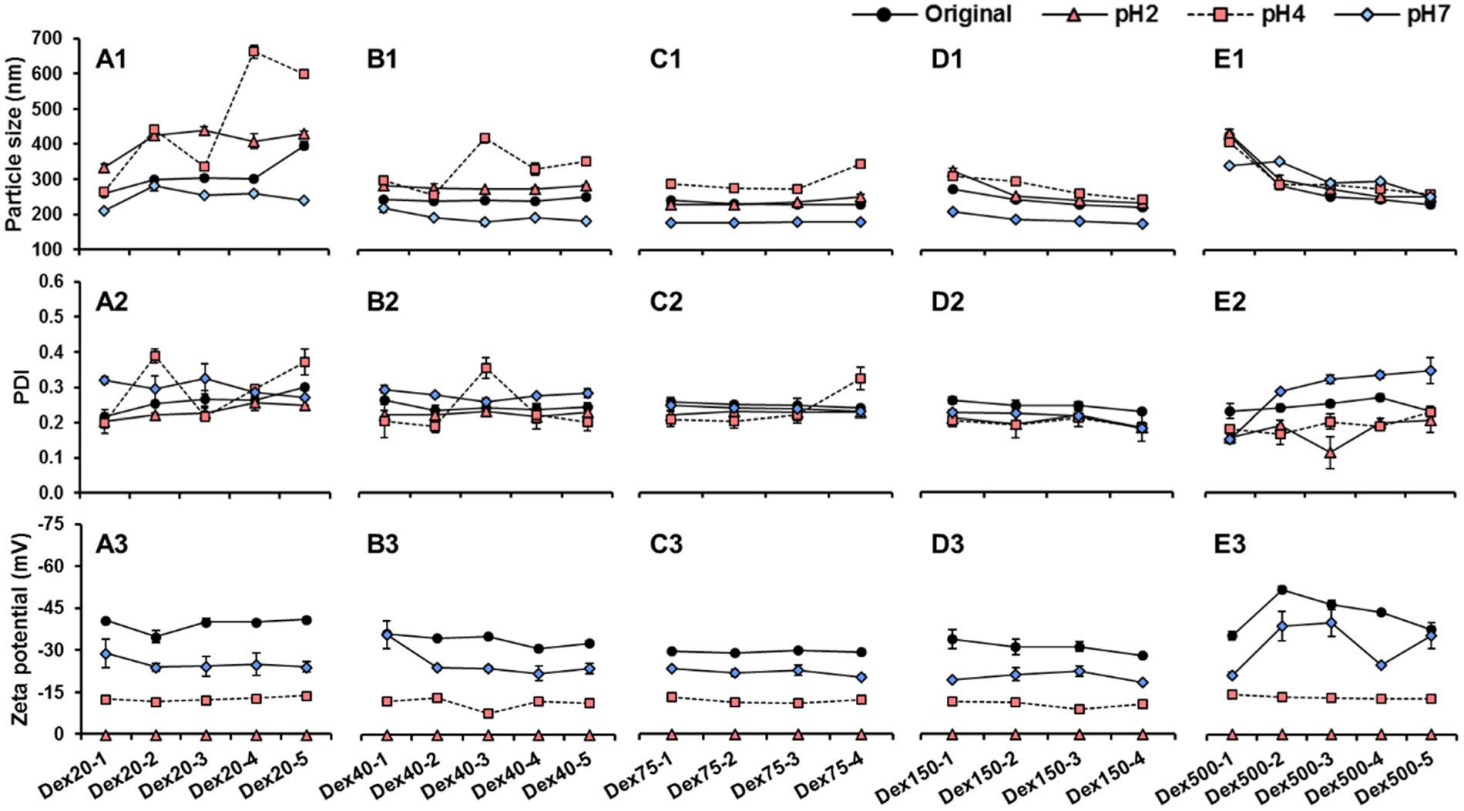
Particle size (A1-E1), PDI (A2-E2), and zeta potential (A3-E3) of LPN in simulated gastrointestinal conditions (pH2, pH4, and pH7).

The gastrointestinal stability of LPN was tested under three simulated digestive environments, i.e. fasting (pH 2) and fed (pH 4) gastric conditions with pepsin, and intestinal condition (pH 7) with pancreatin (Fig. 6). The LPN prepared by the conjugates with either small (20 kDa) or large (500 kDa) molecular weight dextran exhibited poor gastrointestinal stability under all three conditions, showing severe aggregation indicated by large particle size and/or irregular PDI; while those prepared by the conjugates with medium molecular weight dextran (75, 150 kDa) demonstrated excellent stability. The particle size of LPN in the groups Dex75 and Dex150 remained the same or increased slightly at acidic pH, while it significantly reduced by 50 nm at pH 7. This was because the attractive force between the pectin and positively charged protein moieties in the conjugates was well maintained at acidic pHs. But when the pH increased to neutral, their electrostatic attraction was weakened, resulting in the dissociation of pectin coating to some extent and thus reduction in particle size. The change of zeta potential shared the same trend in all LPN samples in the way that its magnitude was gradually reduced as pH decreased and eventually reached zero at pH 2. The stability results reaffirmed that the LPN prepared by BSA-dextran with molecular weight of 75 and 150 kDa were the optimal formulations among all groups.

Although it has been widely reported that the functional properties of Maillard reaction conjugates are dramatically affected by both molecular weight and molar ratio of polysaccharide^39, 40^, our results indicated that the molecular weight of dextran played a predominant role in determining the stability of studied LPN. The carbohydrate tail in the conjugates prepared with small molecular weight of dextran may not be long enough to provide sufficient steric hindrance repulsion to stabilize LPN at acidic pH, leading to the growth of particle size and thus formation of precipitates. Conversely, however, if the molecular weight of dextran is too large (500 kDa), the resultant conjugates may become too hydrophilic, resulting in the reduced emulsifying property, which was also confirmed by the large original particle size of those LPN without pectin coating. It is worth mentioning that either the conjugate or pectin alone was unable to stabilize LPN under pH 2 (data not shown). This suggested that the exceptional stability of pectin-coated LPN at pH 2 with zeta potential being zero was due to the strong steric stabilization effects provided by both BSA-dextran and pectin coating layers. A previous study reported that the BSA-dextran micelles must be cross-linked by glutaraldehyde, a toxic chemical cross-linker, otherwise they would dissociate even at pH 5, a mild acidic condition^31^. Although our previous work demonstrated that chemically cross-linked casein/pectin-coated SLN were able to withstand mild acidic condition (pH 4), they formed larger aggregates and precipitated at pH 2^13^. In summary, the BSA/dextran/pectin-coated LPN prepared in the present study hold promising potential as oral delivery vehicles due to the simple and safe preparation as well as exceptional stability under GI conditions, and thus they are expected to have a better retention of encapsulated bioactives when passing through the GI tract, which warrants future investigation to explore their encapsulation and delivery potentials.

### Fluorescence and FT-IR spectra of LPN

**Figure 7:**
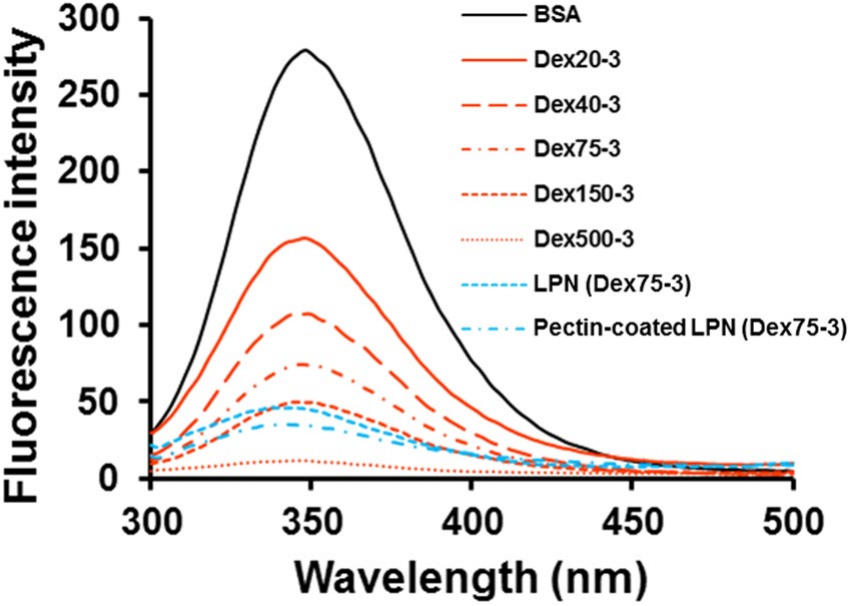
Fluorescence emission spectra of native BSA, selected BSA-Dextran conjugates, LPN and pectin-coated LPN.

BSA is a globular protein with two tryptophan and twenty tyrosine residues, together contributing to its strong intrinsic fluorescence^41^. Maillard reaction of BSA with dextran significantly lowered the intrinsic fluorescence of BSA (Fig. 7), which corresponded to the protein aggregation formed during dry-heating process^28, 42^. Notably, dextran with greater molecular weight exhibited a more significant effect in quenching the intrinsic fluorescence of BSA. Nevertheless, the λmax of BSA when excited at 280 nm did not shift after glycosylation with dextran. This indicated that there was no significant change in the conformation of the protein, which was well corroborated by previous studies on the Maillard reaction between dextran and BSA or other proteins^22, 43^. In contrast, after preparation of LPN and adsorption of pectin, the λ_max_ was significantly shifted from 348 nm to 338 nm, revealing the conformational change in the molecular structure of BSA molecules. However, the degree of conformational change was independent from the molecular weight of dextran (data not shown). It has been reported that heating BSA at or above its denaturation temperature (70 °C) and high-intensity sonication can induce irreversible changes in protein structure and conformation^44, 45^. Since these two processes were involved in the preparation of LPN, it was predictable to observe conformational changes in BSA structure to some extent. The conformational change induced by the heating and sonication process was likely to increase the surface hydrophobicity of BSA-dextran conjugates, which could enhance their capability to stabilize LPN due to the strong hydrophobic interactions.

**Figure 8:**
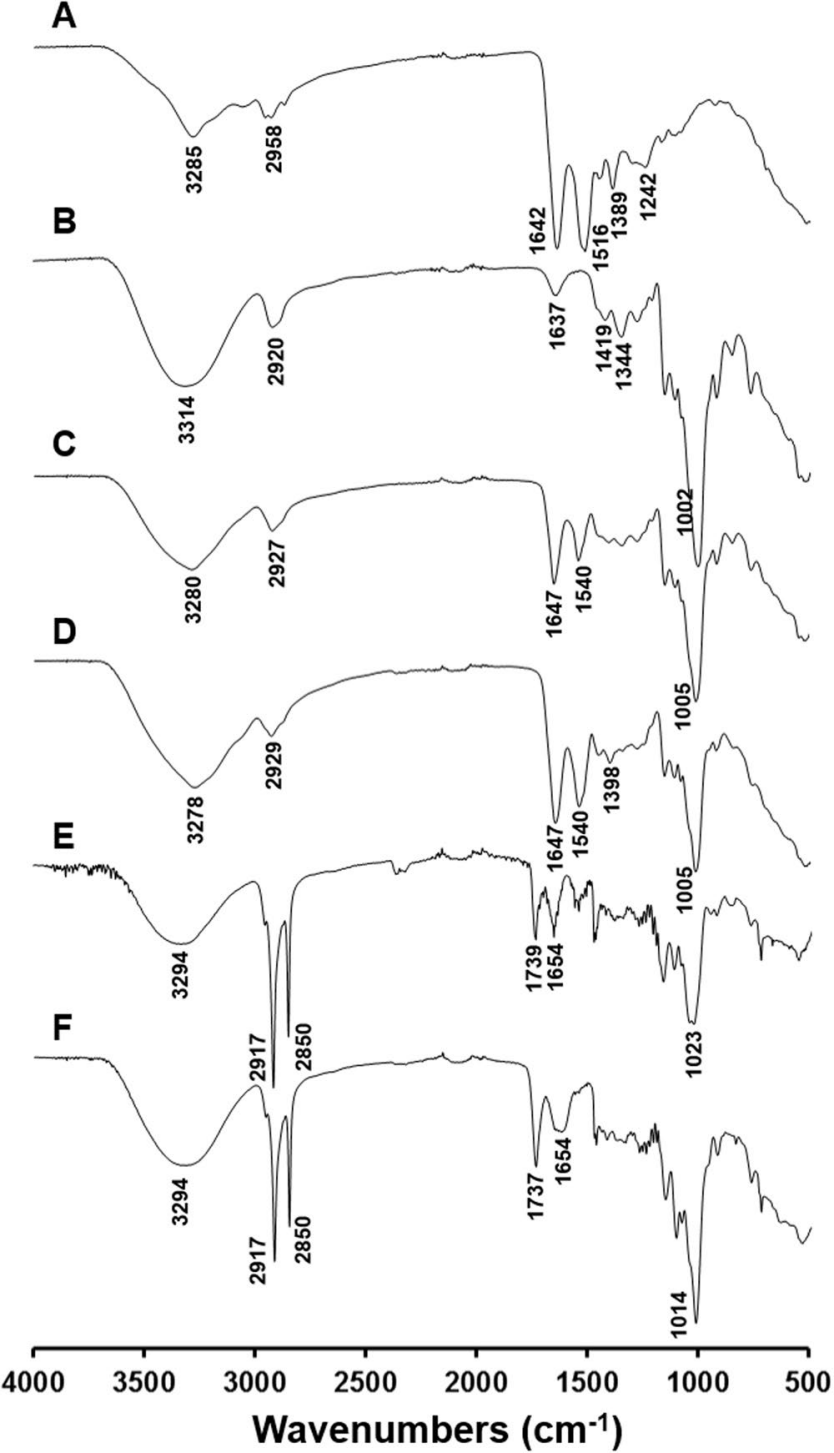
FT-IR spectra of: (**A**) BSA, (**B**) Dextran (M.W = 75 kDa), (**C**) Physical mixture of dextran and BSA, (**D**) Maillard conjugate (Group Dex75-3), (**E**) LPN, (**F**) Pectin-coated LPN.

The FT-IR spectra of all ingredients and LPN are presented in Fig. 8. Two major characteristic peaks at 1642 and 1516 cm^−1^ were observed in native BSA (Fig. 8A) and were assigned to amide I and amide II stretching vibrations, respectively^46^. The dextran spectrum (Fig. 8B) exhibited typical polysaccharide characteristic absorption bands, including 3314 cm^−1^ due to the O‒H stretching, 2920, 1419 and 1344 cm^−1^ assigned to v (C‒H) and δ (C‒H) vibrational modes, as well as a characteristic region at 700–1010 cm^−1^ corresponding to α-glucopyranose ring deformation modes^47^. The physical mixture of BSA and dextran (Fig. 8C) generally exhibited all the characteristic peaks in both compounds. On the contrary, the BSA-dextran Maillard conjugate (Fig. 8D) demonstrated very sharp peaks at the amide stretching region of 1500–1650 cm^−1^, confirming the successful formation of new amide bonds between the two molecules^48^. Dramatic changes were observed in the spectrum of BSA/dextran coated LPN (Fig. 8E), which were attributed to the presence of lipid, a highly hydrophobic compound. In particular, the new peak at 1739 cm^−1^ was assigned to the C=O stretching from carboxyl groups in the saturated aliphatic chain, and the significant reduction of O‒H stretching intensity at 3294 cm^−1^ was owing to the strong hydrophobicity of lipids^25, 49^. It is notable that pectin coating greatly enhanced the hydrophilicity of LPN (Fig. 8F), as suggested by the significant augment in the intensity of O‒H stretching at 3294 cm^−1^ and carboxylate ion vibration at 1014 cm^−1^. These observations were in line with our recent studies on the casein/pectin-coated SLN^12, 13^.

### Morphological observation

**Figure 9:**
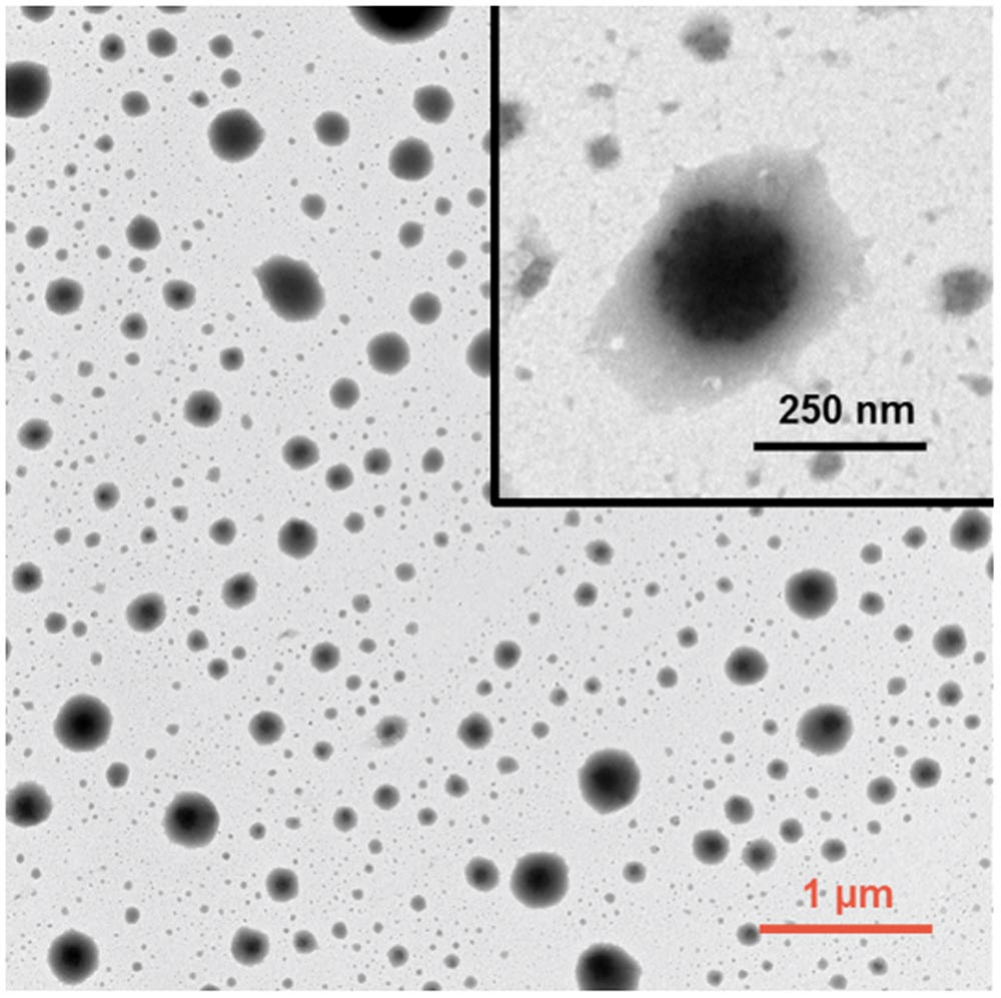
TEM images of pectin-coated LPN (prepared with conjugate Dex75-3).

**Figure 10:**
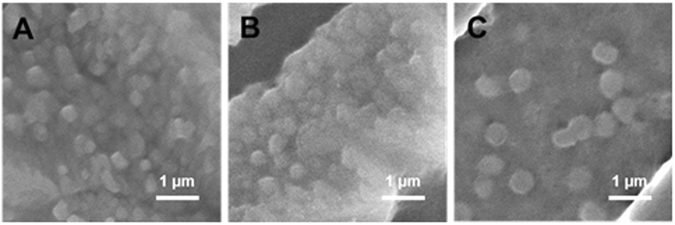
SEM images of liquid samples under pH 2 (**A**), 4 (**B**) and 7 (**C**) of pectin-coated LPN (prepared with conjugate Dex75-3).

TEM was used to image the morphology of pectin-coated LPN prepared with BSA-dextran conjugate Dex75–3. As shown in Fig. 9, the pectin-coated LPN were spherical in shape with monodispersed size distribution, and notably the well-defined core-shell structure was clearly observed, as indicated by strong contrast between the darker core and lighter surface of LPN. The particle size observed from TEM image is in agreement with that determined by DLS (Fig. 5C). Furthermore, the morphology of pectin-coated LPN incubated at different digestive conditions was observed by SEM to verify if there was any morphological change of LPN under each condition. In line with the stability measurement by DLS (Fig. 6), the SEM observation showed that the pectin-coated LPN were able to maintain their spherical shape and homogeneous distribution at all conditions (Fig. 10), including pH 2, 4 and 7 with respective digestive enzymes, implying the successful development of highly stable LPN by a synthetic surfactant-free and cross-linker free synthesis method. It is worth mentioning that the pectin-coated LPN prepared in the present study were even more stable than the covalently cross-linked polymer-coated SLN using chemical cross-linker, such as glutaraldehyde and EDC/NHS reported in other studies^13, 50^.

### Drying and redispersion of pectin-coated LPN

**Figure 11:**
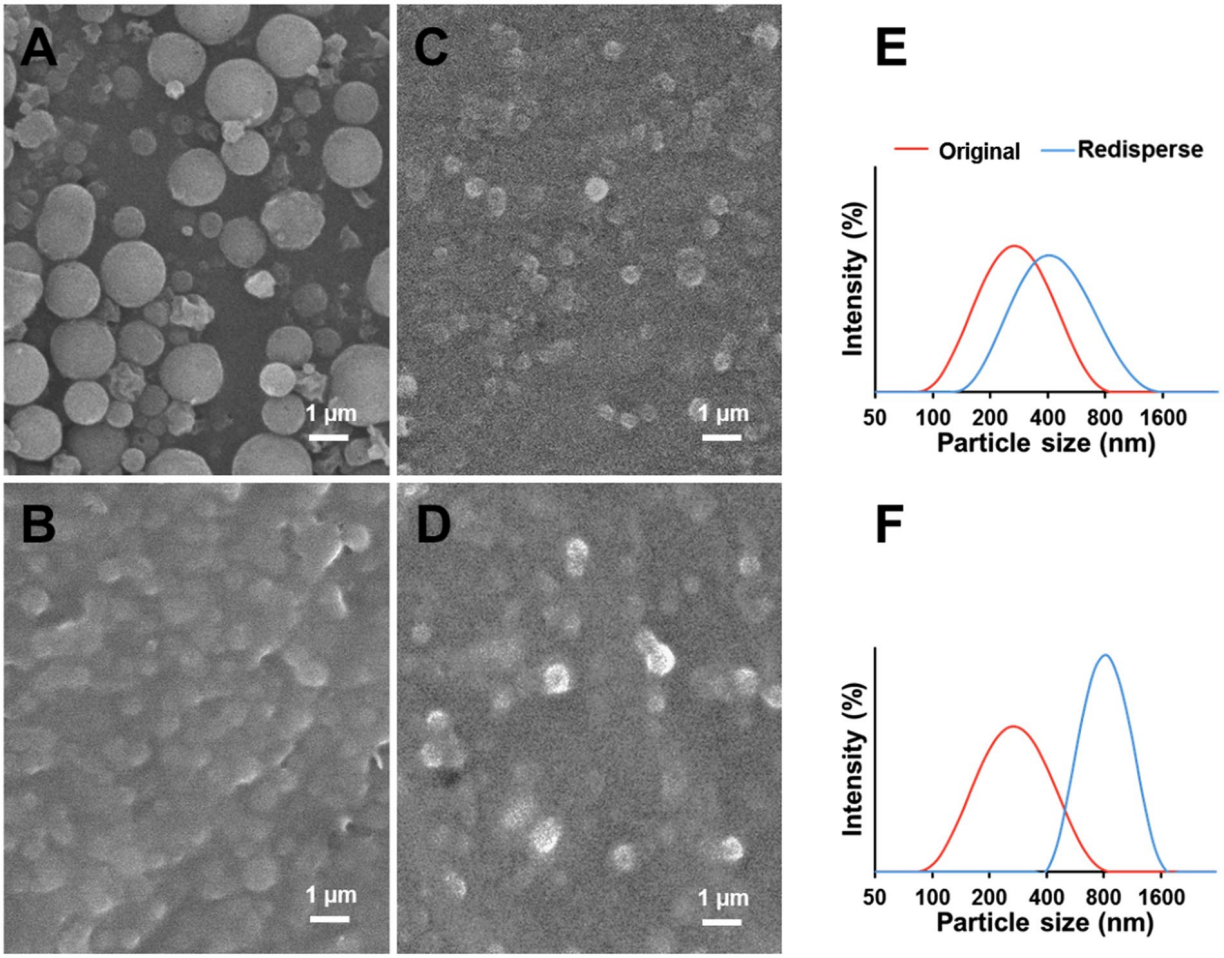
Characterization of dried and redispersed LPN samples: Nano spray-dried (**A**) and freeze-dried (**B﻿**) powders; redispersion of nano spray-dried (**C**) and freeze-dried (**D**) LPN in water; particle size distribution of nano spray-dried (**E**) and freeze-dried (**F**) LPN in water measured by dynamic light scattering.

As shown in Fig. 11A, the Nano Spray Technology transformed pectin-coated colloidal LPN to spherical dry powder particles, with smooth surface and homogeneous size ranging from 1–1.5 µm. Although the particle size was significantly increased in the obtained powders, these powder particles, when redispersed in water, were able to re-assemble their colloidal nanostructures (Fig. 11C), comparing to their original size of 252 nm, suggesting that some irreversible aggregation occurred during drying process. Interestingly, the freeze-dried LPN (Fig. 11B) did not show a significant change in particle size in dry powders and redispersed colloidal LPN (Fig. 11D), both of which were around 500 nm. While the redispersed freeze-dried colloidal LPN were larger than the original LPN, they were much more homogeneously distributed with a dramatically smaller PDI and a narrower distribution curve (Fig. 11E and F).

Spray drying and freeze-drying are the two most commonly used drying techniques to transform colloidal nanoparticles to dry powders. Spray drying technique, characterized by fast water removal rate via heating atomized small droplets at high temperature, is well-known as a simple, dust-free, cost-friendly one-step drying process in food and pharmaceutical industries^51, 52^. Nano Spray Drying technology is a recently invented innovative technology that utilizes a vibrating mesh technology to generate millions of tiny droplets which are efficiently dried in a chamber with laminar hot air flow. In this way, drying time is significantly shortened and minimizes agglomeration of lipid particles. We previously demonstrated the feasibility of this technology to transform caseinate/lecithin and polysaccharides coated colloidal SLN and nanostructured lipid carriers (NLCs) and our results suggested that the biopolymer coatings played a critical role in producing spherical and uniform dry powder particles that were redispersible in water^12–14^. Conversely, freeze-drying technique is a relatively expensive and slow process, and final products are particularly affected by the ice nucleation and crystallization step, i.e. rapid and deep frozen of samples at −80 °C or lower temperature (such as liquid nitrogen) prior to freeze-drying^53^. Despite vast research in obtaining redispersible lipid-based nanoparticles by different drying technologies, many attempts are made with either incorporation of high concentration of sugars (up to 25%) into nanoparticle formulation^54^ or redispersion of dry powders in a solution with concentrated synthetic surfactants^11^. Notably, our results clearly demonstrated that the LPN prepared by using BSA-dextran Maillard reaction conjugate as natural emulsifier together with pectin coating can be dried by nano spray drying and freeze-drying, and the obtained dry powders can redisperse in water with uniform nanoscale size. Nevertheless, it is worth noting that heating at 80 °C for 5 min was needed to fully redisperse the powders without visual presence of precipitates. Therefore, it is postulated that although the LPN may agglomerate into bigger particles during drying, solid lipids were melted upon heating and the polymeric coatings (pectin and BSA-dextran) were re-arranged to the surface of melted lipids which re-assembled to nanoparticles during subsequent cooling process.

## Conclusions

In this work, a synthetic surfactant-free and cross-linker-free technique was developed to prepare highly stable LPN. The formulation consisted of all-natural biomaterials, including solid lipid, BSA-dextran Maillard conjugate, and pectin. The micelle-forming property of BSA-dextran conjugate was exploited to envelop melted solid lipid into the core, followed by an electrostatic deposition of pectin as the outmost polymer coating to reinforce the polymer-lipid network. The molecular weight of dextran and the molar ratio between BSA and dextran were found to be critical in forming compact, uniform, and small LPN with diameter under 200 nm. The as-prepared LPN exhibited exceptional stability under simulated GI conditions with digestive enzymes, as verified by the DLS measurement and SEM observation, signifying their great potential as oral delivery vehicles for lipophilic bioactive compounds.

### Data availability statement

The datasets generated during the current study are included in this published article.
